# Use of Surgical Sponge with Running Sutures for Securing Full-Thickness Skin Grafts

**DOI:** 10.5402/2011/470921

**Published:** 2011-09-29

**Authors:** Stamatis Sapountzis, Achilleas Chantes, Ji Hoon Kim

**Affiliations:** ^1^Department of Plastic and Reconstructive Surgery, Seoul National University Bundang Hospital, 166 Gumiro, Bundang, Gyeonggi, Seongnam 463-707, Republic of Korea; ^2^Plastic Surgeon, 54623 Thessaloniki, Greece

## Abstract

One of the most common methods of skin defect repairing is the use of a skin graft. It is simple and reliable technique, although sometimes it is not totally successful due to hematoma and seroma formation between the skin graft and the recipient bed. Here in, we present a method to secure the skin grafts using a surgical sponge with two running sutures. This technique ensures high survival rate of the skin grafts, and in addition it is easy to be performed by the surgeon only.

## 1. Introduction

The reconstruction of skin defects with skin graft is one of the most common techniques in plastic surgery. It has been proven an especially reliable method. However, skin grafting may be partially, or totally unsuccessful for numerous reasons. The most common cause of skin graft failure is the hematoma formation between the skin graft and the wound bed [1]. 

## 2. Material and Method

**Figure 1:**
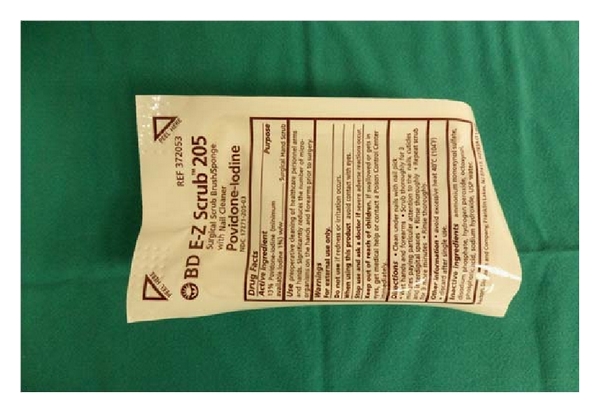
Surgical sponge with Povidone-Iodine.

Here in, we present a technique for full-thickness skin grafts securing, using a surgical scrub sponge, which is saturated with povidone-iodine (Figure 1). Firstly, we secure the skin graft to the recipient bed using a running 5-0 nylon or polypropylene suture. After the skin graft has been sutured to place, we cut the sponge according to the size of the defect—the diameter of the sponge is slight larger than the defect's in order to have a small overlap. Between the sponge and the wound bed we always use a vaseline gauge, in order to have easier removal of the bolster dressing. Then, we perform the “Lilliputian technique” [2] for securing the bolster dressing. We always use two nonabsorbable sutures 4-0 nylon or polypropylene for extra secure in case of the first suture breaks. We remove the dressing bolster 5 to 7 days later.

## 3. Results

**Figure 2:**
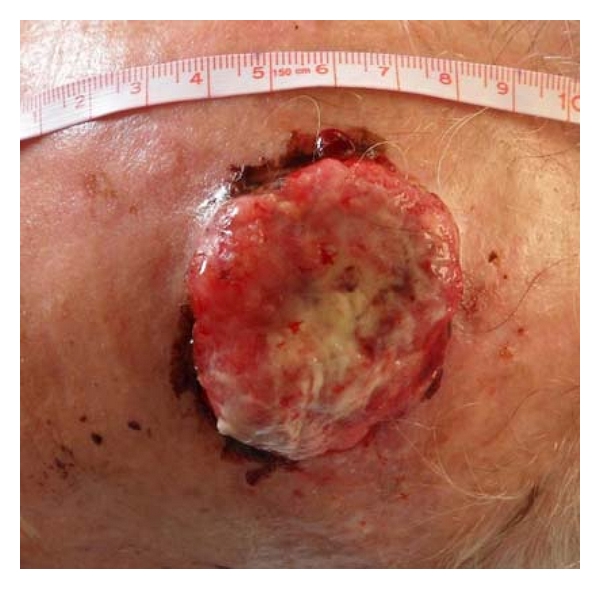
Basal cell carcinoma of the scalp.

**Figure 3:**
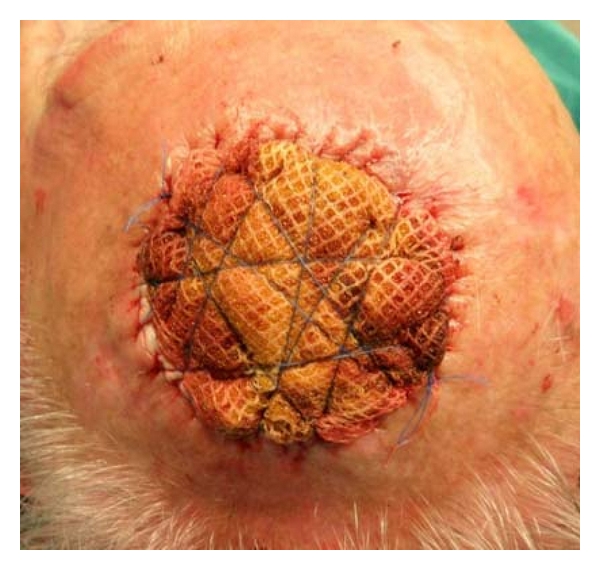
Skin graft secure with surgical sponge and two running sutures.

**Figure 4:**
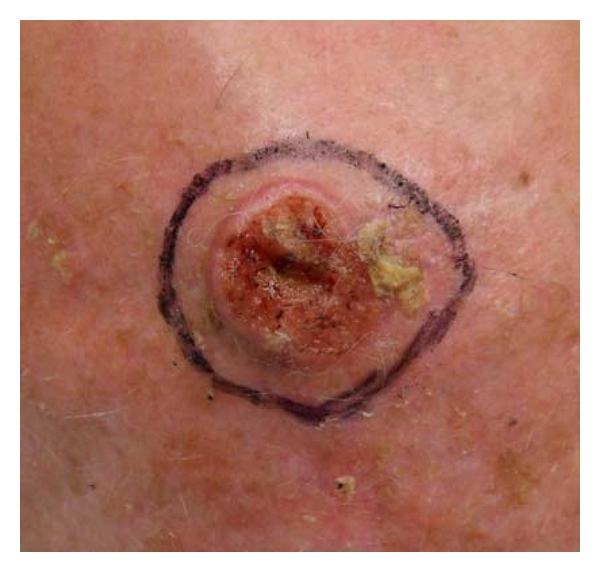
Basal cell carcinoma of the scalp.

**Figure 5:**
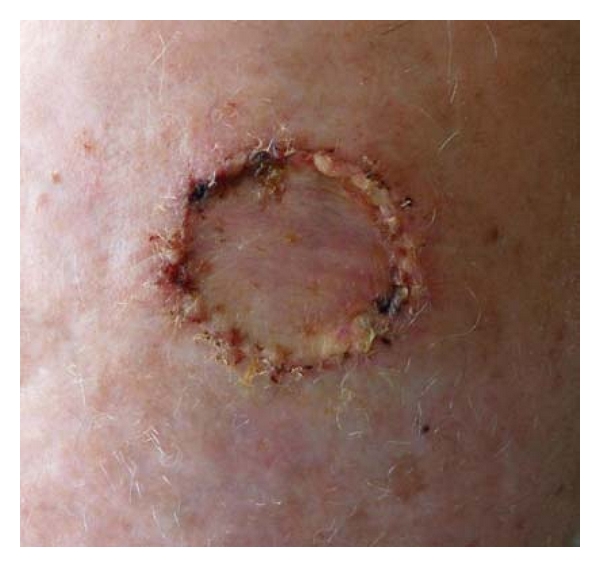
6th postoperative day after the sponge removal.

**Figure 6:**
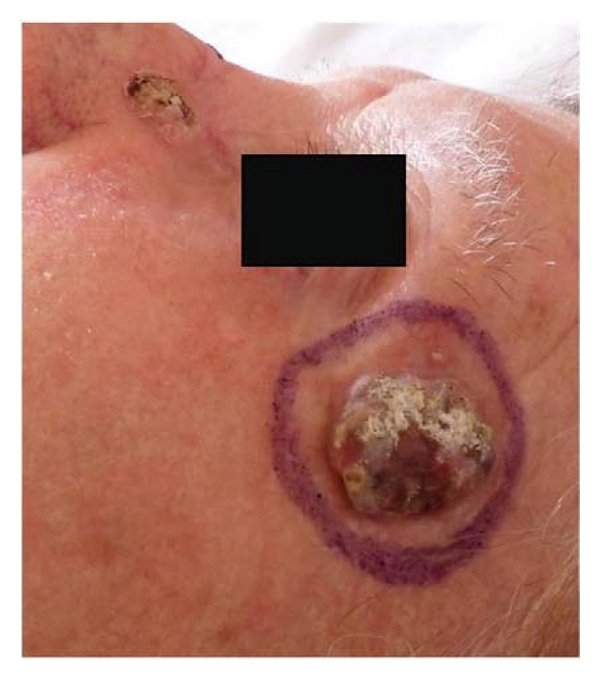
Two epithelioma of the face.

**Figure 7:**
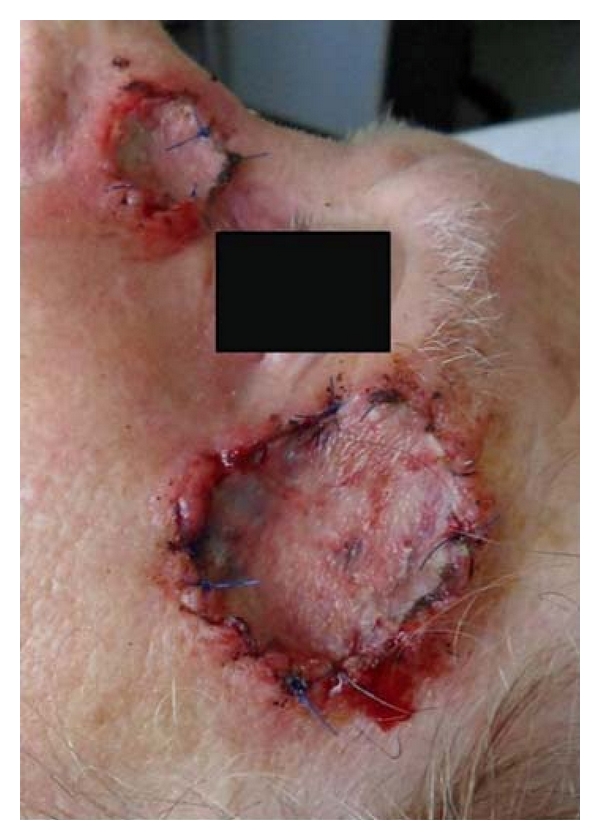
6th postoperative day after the bolster removal.

We performed this technique in 23 patients. All of the skin defects were located in face and scalp, and the mean size was 3.8 cm (2.4–6.1 cm). The mean patient age was 73 years old. The etiology of the skin defect was BCC and SCC excision. In all cases, the percentage of skin graft take was above 90% (Figures 2, 3, 4, 5, 6, and 7).

## 4. Discussion

Many techniques of tie-over dressing for prevention of hematoma and seroma formation have been reported in the literature. The classic “tie-over” dressing consists in multiple interrupted sutures in order to secure the skin graft with the recipient bed. Each stitch's end is intentionally left long, so as to facilitate a tie over a bolster of cotton gauze by joining the loose ends of opposing sutures [3].

Various other bolstering methods include stapled nonadherent gauze [4] staples interlaced with silk sutures [5] and sterile foam compressed with an adhesive dressing [6].

Srivastava and Kouba [2] described a method (“Lilliputian” technique) which involves one or two continuous sutures in order to secure the bolster dressing. Demir at al. [7] also used in their study surgical scrub sponge saturated with Povidone-iodine with success rate in graft take 95.8%.

We prefer to use the surgical sponge because it provides homogenous pressure over the graft. Furthermore, the Povidine-iodine is an anti-infection agent, so it may protect the skin graft from bacterial colonization. Additionally, with the running sutures, it is not necessary to place extra sutures at the center of the graft securing it to the graft bed. The advantage of this technique is that there is no need to reevaluate the skin graft uptake during the first 5–7 days. The pressure of the surgical sponge with continuous sutures ensure the non formation of hematoma and seroma under the skin graft. Furthermore, the technique is easy to be performed by the surgeon only, since there is no need for any assisting hand.

## 5. Conclusion

In our experience, the combination of a surgical sponge with running sutures is an effective, simple, and quick way to secure the skin grafts.

## References

[1] Rudolph P, Ballantyne DL, McCarthy JG (1990). Skin grafts. *Plastic Surgery*.

[2] Srivastava D, Kouba DJ (2009). A “lilliputian” technique for rapid and efficient securing of bolster dressings over full-thickness skin grafts: how we do it. *Dermatologic Surgery*.

[3] Johnson TM, Ratner D, Ratz JL (1997). Skin grafts. *Textbook of Dermatologic Surgery*.

[4] Hoffman HT, La Rouere M (1989). A simple bolster technique for skin grafting. *Laryngoscope*.

[5] Branfman GS, Cassel JM (1988). A simple method for securing a bolster in position over a split-thickness skin graft. *Plastic and Reconstructive Surgery*.

[6] Egan CA, Gerwels JW (1998). Surgical pearl: use of a sponge bolster instead of a tie-over bolster as a less invasive method of securing full-thickness skin grafts. *Journal of the American Academy of Dermatology*.

[7] Demir HY, Tuncer S, Eryilmaz T, Ak B, Ayhan S (2008). A practical tie-over technique: surgical scrub sponge. *Burns*.

